# Tumeur desmoïde géante de la paroi abdominale: à propos d’un cas

**DOI:** 10.11604/pamj.2021.39.211.27965

**Published:** 2021-07-23

**Authors:** Issam Loukil, Amine Zouari

**Affiliations:** 1 Service de Chirurgie Générale Tataouine, Tataouine, Tunisie,; 2 Service de Chirurgie Générale Sfax, Sfax, Tunisie

**Keywords:** Tumeur desmoïde, géante, chirurgie, à propos d’un cas, Desmoid tumor, giant, surgery, case report

## Abstract

La tumeur desmoïde de taille géante rarement rapportée à travers la littérature et qui implique un défi dans sa prise en charge thérapeutique. Pour notre cas la tumeur exerce un effet de masse abdominal, ce qui la rend douloureuse, et un problème esthétique majeur. Le bilan d'extension radiologique a permis de délimiter son extension en profondeur et ses limites d'exérèses. La tumeur desmoïde de la paroi abdominale a été évoquée et réséquée chirurgicalement avec des suites opératoires simples. Ce cas illustre les difficultés de prise en charge de cette entité vu sa grande taille.

## Abstract (en)

Giant desmoid tumor has been rarely reported in the literature but it is a therapeutic challenge. We here report a case of tumor manifesting as painful abdominal mass causing a major esthetic problem. Radiological assessment allowed to determine its depth extension and limits of resection. Desmoid tumor of the abdominal wall was evoked and surgically resected, with simple outcome. This study highlights challenges in the management of this entity,due to its large size.

## Introduction

Les tumeurs desmoïdes sont extrêmement rares. Elles représentent 3% des tumeurs des tissus mous et son incidence est estimée de 2 à 4 cas/millions de population [1]. La taille géante de cette tumeur est rarement rapportée dans la littérature. Le diagnostic positif est difficile et la prise en charge thérapeutique est très complexe nécessitant une approche multidisciplinaire impliquant chirurgien, radiologue et carcinologue, en raison de l´évolution imprévisible et des conséquences fonctionnelles de la maladie [2].

## Patient et observation

**Informations sur le patient**: nous rapportons le cas d´une patiente âgée de 46 ans, sans antécédents pathologiques notables, qui a consulté en juin 2020 pour une volumineuse masse abdominale évoluant depuis plus d´un an.

**Figure 1:**
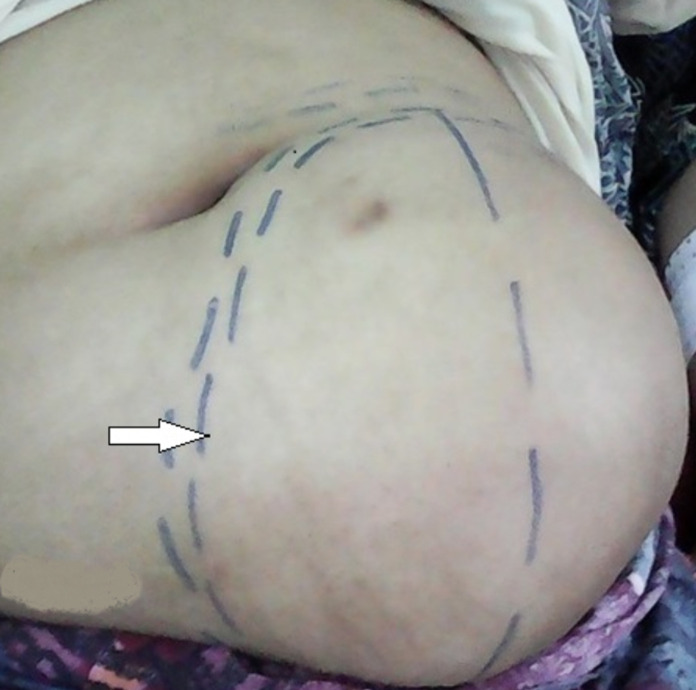
masse abdominale géante, vue préopératoire

**Résultats cliniques**: l´examen a montré une masse de 20 cm de grand axe para-ombilicale gauche, non douloureuse et mobile par rapport au plan profond (Figure 1). La patiente n´a pas rapporté de signes digestifs associés.

**Figure 2:**
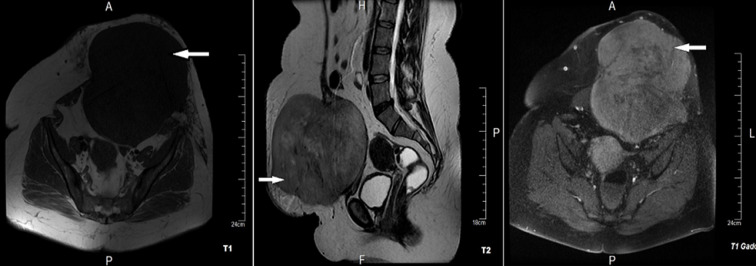
IRM abdominale, masse tissulaire aux dépens du muscle droit de l´abdomen gauche grossièrement ovalaire de contours lobulés mesurant 190x188x160 mm faiblement hypo dense et qui prend le contraste après injection de gadolinium

**Démarche diagnostique**: pour mieux spécifier cette tumeur et étudier ses rapports, un complément d´exploration par imagerie par résonance magnétique (IRM) abdominale en juillet 2020 a montré une volumineuse masse tissulaire aux dépens du muscle droit de l´abdomen gauche grossièrement ovalaire de contours lobulés mesurant 190x188x160 mm faiblement hypo-dense qui prend le contraste après injection de gadolinium (Figure 2). Devant ce tableau une tumeur fibromateuse type desmoïde a été évoquée et une colonoscopie a été demandée à la recherche de polypes coliques associés, revenue sans anomalies.

**Figure 3:**
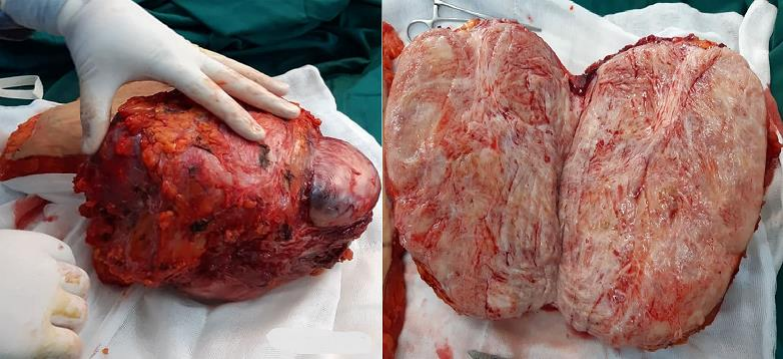
aspect macroscopique de la masse abdominale réséquée

**Figure 4:**
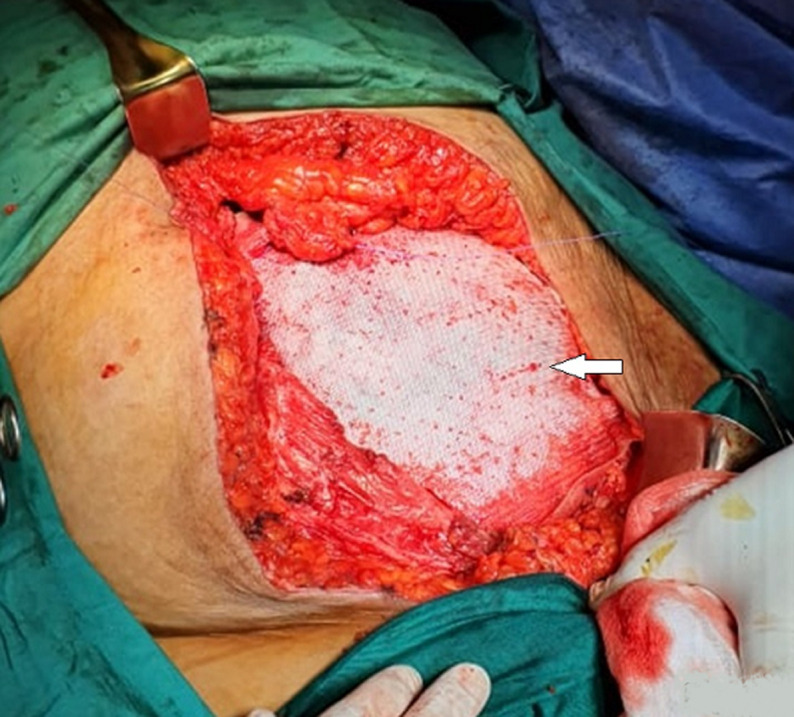
réfection du défect pariétal par une prothèse synthétique biface

**Intervention thérapeutique**: la patiente a été opérée par laparotomie médiane. L´exploration peropératoire montre une masse polylobée aux dépends des différentes tuniques adjacentes de la paroi abdominale gauche sans extension ni localisations secondaires intra-péritonéale. Une résection en monobloc de cette masse a été faite emportant le plan cutané, sous cutané, la partie musculaire envahie ainsi que ses aponévroses et le plan péritonéal adjacent avec une marge chirurgicale macroscopique de sécurité minimale de 1 cm en latéral et en profondeur (Figure 3). La fermeture pariétale a nécessité un geste de reconstruction par une prothèse synthétique biface (Figure 4).

**Figure 5:**
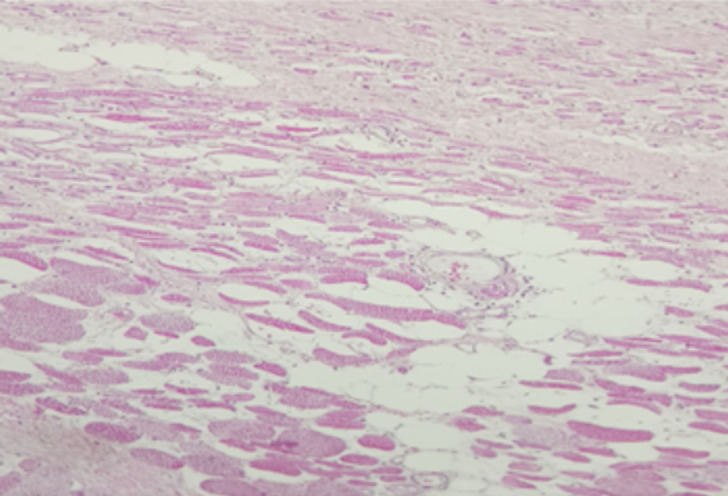
aspect histologique

**Suivi et résultats des interventions thérapeutiques**: l´étude histologique de la pièce opératoire a confirmé le diagnostic d´une fibromatose agressive en montrant un agencement cellulaire en longs faisceaux divergents dissociant le muscle strié avec marge de résection microscopique saine (Figure 5). Le dossier a été présenté en réunion de concertation pluridisciplinaire et un traitement adjuvant postopératoire n´a pas été indiqué. Un contrôle à 6 mois clinique et scannographique n´a pas montré de signes de récidive locale.

**Perspectives du patient**: la patiente a déclaré être satisfaite de la qualité de la prise en charge et des soins fournis.

**Consentement éclairé**: la patiente a fourni son consentement.

## Discussion

Le point fort de cette publication est qu´il rapporte un cas très rare de tumeur desmoïde de grande taille. Notre approche s´est basée sur l´étude des cas rapportés, bien que limités à travers la littérature et notre discussion du consensus de prise en charge. La tumeur desmoïde est définie par l´Organisation Mondiale de la Santé en tant qu´une prolifération fibroblastique clonale qui survient dans les tissus mous profonds et se caractérise par une incapacité à métastaser même si elle peut être multifocale dans le même membre ou partie du corps [3]. Elle touche plus fréquemment les femmes entre 15 et 60 ans avec un pic entre 30 et 40 ans [4,5].

**Deux grandes catégories de tumeurs desmoïdes sont reconnues**: les tumeurs sporadiques majoritaires de 85 à 90 % des cas associée à une mutation dans la voie codante pour la béta-caténine CTNNB1, et les tumeurs associées à la mutation du gène APC de la polypose adénomateuse familiale (PAF) touchant principalement l´abdomen [4,6]. Le tableau clinique peut présenter plusieurs aspects selon la localisation et peuvent être très agressives avec une croissance accrue et effet de masse comme pour le cas de notre patiente [7]. L´IRM est l´examen de référence pour le diagnostic, la stadification et le suivi. Elle permet d´étudier l´extension tumorale et de déterminer le plan de résection chirurgicale à atteindre. Sur les images pondérées en T1, la tumeur apparait hypo ou iso-intense par rapport au muscle et apparait hyper-intense en T2 avec un renforcement après injection de gadolinium et persistance de bondes hypo-intenses [3,4]. Le diagnostic de certitude est histologique sur une biopsie percutanée ou de la pièce opératoire en montrant une prolifération de cellules fusiformes uniformes ressemblant à des myofibroblastes, dans un stroma de collagène abondant et de réseau vasculaire.

**La prise en charge thérapeutique comporte plusieurs volets**: chirurgical, systémique et locorégional. Le traitement chirurgical reste un défi pour les chirurgiens surtout pour les tumeurs géantes. En raison du schéma de croissance infiltrant, la portée de la résection nécessaire pour obtenir des marges négatives pourrait souvent entrainer des altérations fonctionnelles importantes et des altérations esthétiques [5]. Cette résection confère un bon taux de contrôle local à environ 80% à 5 ans [3]. Aucun consensus concernant la technique chirurgicale de fermeture des défects abdominaux n´a été établit. Le chirurgien aura le choix entre l´utilisation des prothèses pariétales synthétiques ou l´utilisation de lambeaux libres musculo-cutanés et les procédés de rapprochement aponévrotiques [1]. La chimiothérapie reste une option dans les cas des maladies symptomatiques non résécables ou avancées [4-6]. Les thérapies ciblés (Imatinib) confère un taux élevé de stabilisation de la maladie de 60 à 80% des cas [5], l´hormonothérapie (Tamoxifène) par son action anti-oestrogéniques est associée à un bénéfice clinique dans environ 30% des cas [6] et les anti-inflammatoire non stéroïdien (AINS) par leur capacité de réguler la voie de la ß-caténine par l´inhibition de la cyclo-oxygénase-2 ou des prostaglandines [5]. Le traitement locorégional par radiothérapie ou de cryothérapie peut réduire le risque de récidive après résection chirurgicale incomplète [3,4,6,8].

**Figure 6:**
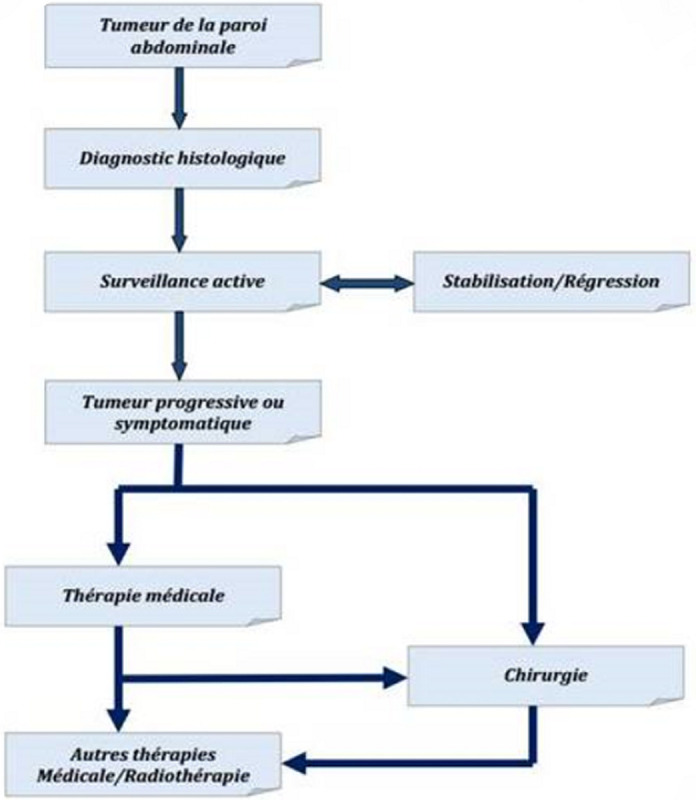
algorithme de prise en charge

Les recommandations du consensus de Milan 2018 ont défini un algorithme de prise en charge (Figure 6) et adopte la stratégie initiale de surveillance active pour les patients qui présentent des tumeurs desmoïdes pour une période de 1 à 2 ans par examen clinique et IRM mensuel durant les premiers mois puis à un intervalle de 3 à 6 mois, à la recherche de progression tumorale ou augmentation des symptômes qui va justifier un traitement actif [4,9]. Certaines situations rendent la chirurgie inévitable telle que les complications (occlusions, perforation, hémorragie) ou des problèmes esthétiques majeurs, tel est le cas de notre patiente. La surveillance après résection chirurgicale, est essentiellement clinique et radiologique par une IRM ou tomodensitométrie (TDM) [10]. Les taux de récidive locale dépendent essentiellement des marges de résection. Les tumeurs de grandes tailles sont plus susceptibles de récidiver. En conclusion, il s´agit bien d´une tumeur rare dont la taille géante rend sa prise en charge plus délicate. L´approche thérapeutique actuelle opte pour la stratégie de surveillance active. Le cas de notre patiente présente des arguments d´atteinte esthétique et de douleur abdominale, indiquant une résection chirurgicale d´emblée. La résection de cette masse implique un deuxième geste de réfection pariétale par plaque synthétique.

## Conclusion

Les tumeurs desmoïdes géantes sont des fibromatoses agressives du fait de leur caractère infiltrant avec un haut potentiel de récidive. Le diagnostic doit être évoqué devant toute masse tissulaire pariétale abdominale. L´IRM permet de poser le diagnostic, de guider la prise en charge thérapeutique et de suivre l´évolution. La confirmation du diagnostic est histologique. Le traitement repose actuellement sur une stratégie de surveillance active initiale en quête de signes de complications ou de progression.
